# Supplementary material for: Predictors of DAPSA Response in Psoriatic Arthritis Patients Treated with Apremilast in a Retrospective Observational Multi-Centric Study
Source: Biomedicines. 2023 Feb 2;11(2):433. doi: 10.3390/biomedicines11020433 (PMC9953385; doi:10.3390/biomedicines11020433)
Supplement: Supplementary file 1 [file biomedicines-11-00433-s001.zip › biomedicines-2165547-supplementary.pdf]

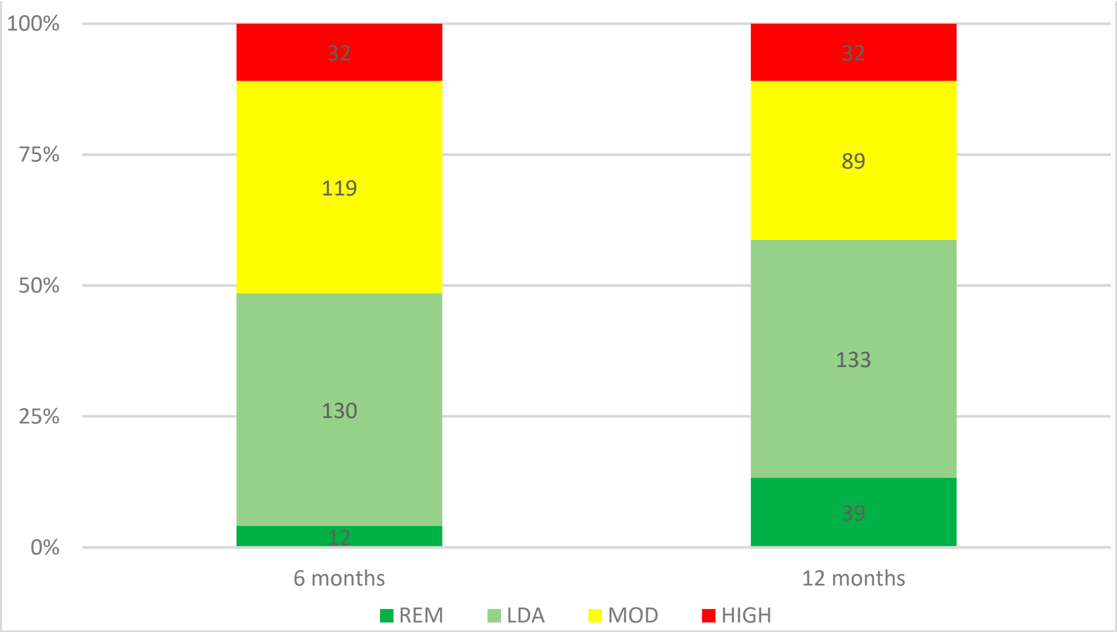

Supplementary Figure S1. cDAPSA scores at 6 and 12 months. REM remission, LDA low disease activity, MOD moderate disease activity, HIGH high disease activity.

Supplementary Table S1. Univariate and multivariate analysis of variables associated with achivement of cDAPSA low disease activity and remission at 6 months.

	Univariate Analysis			Multivariate Analysis		
Variable	OR	95% CI	p	OR	95% CI	p
Age	0.987	0.968 – 1.006	0.799			
Smoke	1.039	0.549 – 1.968	0.905			
Sex	1.178	0.741 – 1.871	0.488			
BMI	0.991	0.941 – 1.044	0.738			
Relevant comorbidity	1.52	0.958 – 2.412	0.075	1.943	1.132 – 3.336	0.016
Disease duration	0.999	0-996 – 1.003	0.688			
Baseline DAPSA	0.883	0.852 – 0.915	<0.0001	0.881	0.849 – 0.913	<0.0001
Concomitant csDMARD	0.764	0.428 – 1.364	0.362			
Number of previous bDMARD	0.711	0.564 – 0.895	0.0036	0.779	0.614 – 0.989	0.0407
Number of previous csDMARD	1.046	0.814 – 1.344	0.726			

Supplementary Table S2. Univariate and multivariate analysis of variables associated with achivement of cDAPSA low disease activity and remission at 12 months.

	Univariate Analysis			Multivariate Analysis		
Variable	OR	95% CI	p	OR	95% CI	p
Age	1.007	0.9877 – 1.027	0.887			
Smoke	0.972	0.510 – 1.852	0.932			
Sex	1.305	0.813 – 2.095	0.269			
BMI	0.986	0.935 – 1.039	0.598			
Relevant comorbidity	1.337	0.837 – 2.137	0.224			
Disease duration	1.002	0.998 – 1.005	0.359			
Baseline DAPSA	0.933	0.908 – 0.959	<0.0001	0.935	0.909 – 0.961	<0.0001
Concomitant csDMARD	1.283	0.708 – 2.325	0.412			
Number of previous bDMARD	0.808	0.664 – 0.984	0.0336	0.843	0.688 – 1.034	0.101
Number of previous csDMARD	1.202	0.926 – 1.562	0.167			
